# The QUIT-PRIMO provider-patient Internet-delivered smoking cessation referral intervention: a cluster-randomized comparative effectiveness trial: study protocol

**DOI:** 10.1186/1748-5908-5-87

**Published:** 2010-11-17

**Authors:** Thomas K Houston, Rajani S Sadasivam, Daniel E Ford, Joshua Richman, Midge N Ray, Jeroan J Allison

**Affiliations:** 1 Center for Health Quality, Outcomes & Economic Research (CHQOER), Bedford VAMC, Bedford, MA, USA; 2 VA eHealth Quality Enhancement Research Initiative, Bedford VAMC, Bedford, MA, USA; 3 Division of Health Informatics and Implementation Science, Quantitative Health Sciences and Medicine, University of Massachusetts Medical School, Worcester, MA, USA; 4 The Johns Hopkins University School of Medicine, Baltimore, MD, USA; 5 School of Medicine, University of Alabama at Birmingham, Birmingham, AL, USA; 6 School of Health Professions, University of Alabama at Birmingham, Birmingham, AL, USA

## Abstract

**Background:**

Although screening for tobacco use is increasing with electronic health records and standard protocols, other tobacco-control activities, such as referral of patients to cessation resources, is quite low. In the QUIT-PRIMO study, an online referral portal will allow providers to enter smokers' email addresses into the system. Upon returning home, the smokers will receive automated emails providing education about tobacco cessation and encouragement to use the patient smoking cessation website (with interactive tools, educational resources, motivational email messages, secure messaging with a tobacco treatment specialist, and online support group).

**Methods:**

The informatics system will be evaluated in a comparative effectiveness trial of 160 community-based primary care practices, cluster-randomized at the practice level. In the QUIT-PRIMO intervention, patients will be provided a paper information-prescription referral and then "e-referred" to the system. In the comparison group, patients will receive only the paper-based information-prescription referral with the website address. Once patients go to the website, they are subsequently randomized within practices to either a standard patient smoking cessation website or an augmented version with access to a tobacco treatment specialist online, motivational emails, and an online support group. We will compare intervention and control practice participation (referral rates) and patient participation (proportion referred who go to the website). We will then compare the effectiveness of the standard and augmented patient websites.

**Discussion:**

Our goal is to evaluate an integrated informatics solution to increase access to web-delivered smoking cessation support. We will analyze the impact of this integrated system in terms of process (provider e-referral and patient login) and patient outcomes (six-month smoking cessation).

**Trial Registration:**

Web-delivered Provider Intervention for Tobacco Control (QUIT-PRIMO) - a randomized controlled trial: NCT00797628.

## Background

Tobacco use is the number one behavioral health problem and number one preventable cause of death [1-5]. Interventions to reduce smoking have most frequently targeted patients. Patient self-management interventions for smoking cessation include mass dissemination of tobacco cessation self-help materials, computer-tailored printouts, interactive voice response systems, and more recently, "quitlines" and smoking cessation websites [3,6-13]. Unfortunately, self-management interventions for smoking cessation have been underutilized. Studies of quitlines note that as little as 3.5% of adult smokers call per year [14]. Because the majority of smokers (70%) see a healthcare provider at least once per year [15], physician referrals could greatly increase use of publicly available self-management interventions for smoking.

Quality improvement and implementation interventions have tried to change processes of care or provider behavior related to tobacco control with some success. Brief clinical interventions, based on tobacco use screening and brief, structured cessation advice from a provider, have been documented to improve patient cessation rates [15-18]. The current US Department of Health and Human Services clinical practice guideline entitled "Treating Tobacco Use and Dependence" provides a summary of evidence-based recommendations [5]. The current guideline includes a framework for structured, brief clinical interventions using the "5 *A*s" of counseling:

1. Ask: Identify and document tobacco use status for every patient at every visit.

2. Advise: In a clear, strong, and personalized manner, urge every tobacco user to quit.

3. Assess: Is the tobacco user willing to make a quit attempt at this time?

4. Assist: Refer to resources and provide pharmacotherapy and counseling.

5. Arrange: Schedule follow-up contact, preferably within the first week after the quit date.

The first two *A*s (Ask and Advise) have increased through system-based interventions (*i.e*., smoking status as a vital sign) [19,20] and audit and feedback of smoking counseling performance [21,22]. However, implementation of Assist and Arrange has been lower [21,23,24]. One important component of assisting patients mentioned in the guideline is to refer patients to community resources, such as quitlines [15]. As quitlines and websites have proliferated, "Refer," as part of the Assist agenda, has been increasingly emphasized [25]. In practice, rates of referral to cessation resources have been measured to be as high as 28% at the VA [26] and 37% in managed care [27] and as low as 10% in community-based practices [28]. Providers do refer some patients to quitlines. In one study, 20% of quitline users were referred by providers [29]. Barriers to Refer include provider's lack of (a) time due to competing demands, (b) awareness of referral resources, (c) prompts, (d) materials to facilitate referrals, and (e) feedback on referral's success [30]. Both patient and provider barriers to using resources for smoking cessation could potentially be addressed with an integrated system.

In this report, we describe the protocol for the QUIT-PRIMO-quality improvement in tobacco-provider referrals and Internet-delivered microsystem optimization-provider-to-patient informatics system. The informatics system will allow providers at the point of care to use a simple web portal to "e-refer" patients to a smoking cessation website. Providers simply type the smokers' email into the ReferaSmoker. Patients will then receive motivational emails encouraging them to join the patient intervention website (with interactive tools, educational resources, motivational email messages, secure messaging with tobacco treatment specialists and an online support group). Providers will subsequently get reports of patient activity on the smoking cessation website.

Our overall goal is to advance science related to the use and impact of the Internet in health services delivery of tobacco control. Because the informatics system is designed to engage all providers in a primary care clinic, including physicians and nurses, we will evaluate the system in a cluster-randomized trial. We will randomize 160 primary care clinical microsystems to the intervention or comparison group. For both the intervention and comparison groups, we will adapt protocols used in prior successful Internet-delivered provider interventions to recruit practices and implement the system in practices [31,32]. Because our trial targets both practices and patients, patients within practices undergo a second level of randomization, as described below. We will use the discussion of our cluster-randomized trial to detail our approach to inherent measurement challenges in this randomized trial of a mixed provider-patient informatics intervention.

## Methods

### Study design

We will recruit 160 primary care physician practices to our trial. As further detailed below, our primary intervention target is the clinical practices. Patients nested within these practices will be cluster-randomized to receive either a simple paper referral or the full intervention-a paper referral plus an "e-referral" (smoker's email will be entered into a referral system and the smoker will receive encouraging emails to participate). In addition to our primary cluster-randomized trial, patients who participate in the website will be further randomized (patient-level, within-practice randomization) to receive either a standard or augmented patient website. Thus, our design is a randomized trial of a patient smoking cessation intervention, nested within a cluster-randomized practice intervention.

### Participants

Our target is the primary care clinical microsystem within family practice and general internal medicine practices from across the United States. A clinical microsystem is defined as the smallest functional healthcare unit. A clinical microsystem is not simply equivalent to a group of doctors, but includes the clinical team of nurses, the processes of care that are used, and the panel of patients cared for by the providers. The Institute for Healthcare Improvement states that interventions targeted to clinical microsystems are "a crucial component in improving health care quality." [33] Our informatics intervention targets both the practice staff and their patients, and thus, both practices and patients are described as participants.

#### Participating practices

Practices will be recruited using a database of registered internal medicine and family/general practitioners. Initial interest will be ascertained via mass mailing of an interest survey. Once initial interest is expressed by return of the brief interest survey, each practice will be assessed for inclusion.

We are including community-based primary care practices (general internal medicine and family practice). Exclusion criteria include those practices that do not have Internet access available to staff and practices that do not see at least five or more smokers in one week. In addition, we will selectively recruit practices that have five or less providers. Based on our prior experience, enrollment of practices in these studies is complex and is somewhat easier if the number of providers in the practice is lower. Thus, we will exclude practices that have greater than five physicians. We will also exclude practices with ongoing computer-based smoking cessation programs, and we will not recruit from practices participating in ongoing studies or similar prior studies, especially those focused on tobacco control.

#### Participating patients

Patients will be referred to an online smoking cessation system (Decide2Quit.org). Patients referred will be adult smokers in the intervention and comparison practices. Decide2Quit.org is designed as a cessation induction and support for quitting system, tailored to readiness to quit. Thus, we are including those ready to quit, thinking about quitting, or not thinking about quitting.

### Interventions

Our discussion of the interventions begins with a comparison of the practice-level intervention.

#### ReferaSmoker.org, the practice-level intervention

**Figure 1:**
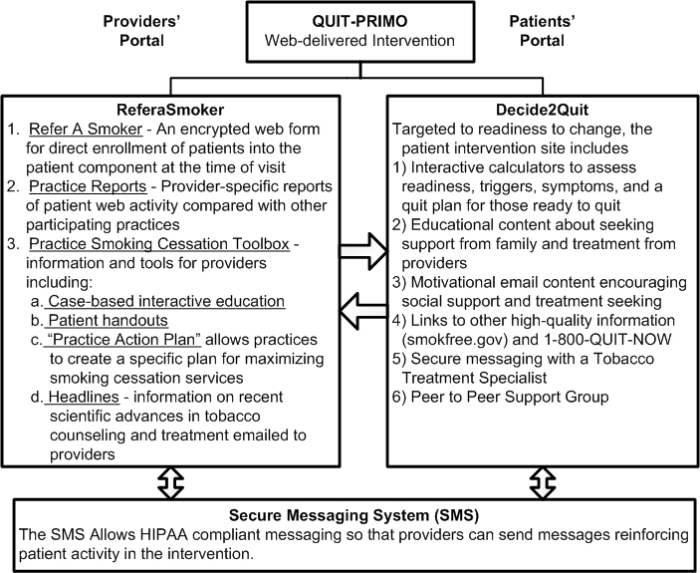
Major components* of QUIT-PRIMO provider-patient informatics intervention. * All components are supported by repeated, targeted email reminders designed to prompt participation and cue increased smoking cessation. Emails will invite enrolled smokers, provide motivational and educational messages to enrolled smokers, notify providers of new web reports, and alert providers to new messages from patients. A proactive Help Desk will also be available as part of the intervention.

Intervention practices will be provided preprinted pads of "information prescriptions" with their office information, a space for the provider to sign, and the smoking cessation website address (Decide2Quit.org). The information prescriptions are perforated; half will be retained by the practice (including the patient email to be used for e-referral) and half will be provided to the patient (see Additional File 1, Appendix 1). Practices will then use this patient email collection to e-refer them through ReferaSmoker.org. The core of the ReferaSmoker.org provider portal is a secure sockets layer (SSL) encrypted web form where providers can enter patients' email addresses into the system if they agree to be referred (Figure 1). The form has been designed to be easily completed by nursing or front office staff as the patient is discharged from the visit. Online referrals through the SSL form will be tracked by the server. The practice-reports function will provide feedback reports to providers on their patients' progress and their practice's referral rates. These feedback reports will act as a proximal outcome, where providers of all types can actually observe the impact of their efforts. To maximize the use of ReferaSmoker.org, we also provide supportive sub modules designed to prompt providers to use the system and maximize their smoking cessation activities (Figure 1, ReferaSmoker #3).

#### ReferaSmoker.org implementation program

After practices are enrolled, we will then schedule and conduct individualized telephone trainings with two implementation coordinators (physicians, nurses, or other staff) chosen at each practice. We chose two implementation coordinators per practice because of high rates of turnover of office staff in our prior experiences. Two coordinators can provide each other backup and further enhance use of ReferaSmoker.org. Using an academic detailing approach, our study team will walk the implementation coordinators through the ReferaSmoker website, including initial registration, and practice e-referring a test patient.

Using motivational interviewing, we will work with the implementation coordinators to identify barriers and strategize solutions to enhance participation. Implementation coordinators will set a goal for number of referrals per week based on their practice volume. Implementation coordinators will be trained on registering other providers in the practice into the system.

Based on pilot testing and focus groups, we have identified specific incentives, including provision of continuing education credits to participating providers and a $1,000 per practice "implementation budget" for completing training and referring their first 20 smokers. Proactive booster calls will be scheduled one month after initial registration to assess progress, respond to any questions, and continue to motivate participation.

#### The practice-level comparison

The practice-comparison referral process ends at the information prescriptions. Comparison practices will be enrolled in the same manner and will participate in the implementation program training calls. Randomization will occur on the calls once registration is complete, as further described below. The ReferaSmoker.org portal changes dynamically based on the randomization status of the practice, and comparison practices will only receive the supportive educational materials (Figure 1, ReferaSmoker #3). Comparison practices do not have access to the e-referral system, the practice feedback dashboard, or the secure messaging system.

As noted, comparison practices are provided preprinted pads of information prescriptions, exactly like those the intervention practice received, save one detail. There is no space for the patient email because control providers will not use the e-referral system (see Additional File 2, Appendix 2). Smokers will be provided half the information prescriptions, with the Decide2Quit.org address, just as with the intervention practices. All other components of the implementation program, motivational interviewing and goal-setting, incentives, and booster calls are kept constant across the two arms. Practices randomized to the comparison do receive a more limited training, focusing on the paper information prescriptions.

#### Decide2Quit.org, the patient-level intervention

Providers refer patients to Decide2Quit.org by paper prescription in the comparison group or by paper plus e-referral in the intervention group. Patients e-referred to Decide2Quit.org will receive reminder emails (two per week for four weeks) as cues to participation.

Patients who follow the referral and register with Decide2Quit.org will complete an online consent form and a baseline survey, including assessment of their readiness to quit smoking. This baseline data will be used to tailor the website to the individual.

Once registration is complete, smokers from both intervention and control practices will be further randomized. This within-practice randomization will allow smokers to receive one of two versions of Decide2Quit.org: a standard Decide2Quit.org or an augmented version of Decide2Quit.org (Table 1).

**Table 1:** Major components of Decide2Quit.org

Component	Description
MyMail^a^	Receive messages from a tobacco treatment specialist

Our Advice^a^	Receive encouraging email messages from experts; messages tailored to stage of change

Your Online Community^a^	View messages and dialogue from smokers and ex-smokers through a resource website

My Health Risks^b^	Learn about specific health risks, including physical symptoms and harmful chemicals

Thinking About Quitting^b^	Helpful ideas and motivational recommendations (*e.g*., interactive calculators assessing triggers, decisional balance)

Family Tools^b^	How to get help from your family, deal with nagging, learn what kids think about smoking

Healthcare Provider Tools^b^	How to include your healthcare provider in your quit smoking plan

The Library^b^	Download articles and helpful tools about smoking cessation and smoking treatments

Web Resources^b^	Valuable additional websites for smokers

^a^These components are available only to the augmented Decide2Quit.org intervention; ^b^Standard components available to all smokers registered to Decide2Quit.org, both those randomized to standard version and those randomized to augmented version.

The standard comparison Decide2Quit.org includes a library of information about quitting smoking, including educational content about talking to a doctor about quitting smoking and detailed information on medications and behavioral treatments. The system also includes content about seeking help from friends and family, a chemicals-in-smoking matching game, and a decisional-balance "what will I have to overcome" calculator with individualized feedback. Smokers can complete a personalized "Quit Plan" that they can print and share with their provider.

The augmented Decide2Quit.org intervention includes all the components of the standard intervention plus (a) pushed motivational emails tailored to readiness to quit and designed to motivate cessation and market the Decide2Quit.org intervention; (b) secure asynchronous messaging with a personal advisor, a trained tobacco treatment specialist; and (c) an online support group community (Table 1).

**Figure 2:**
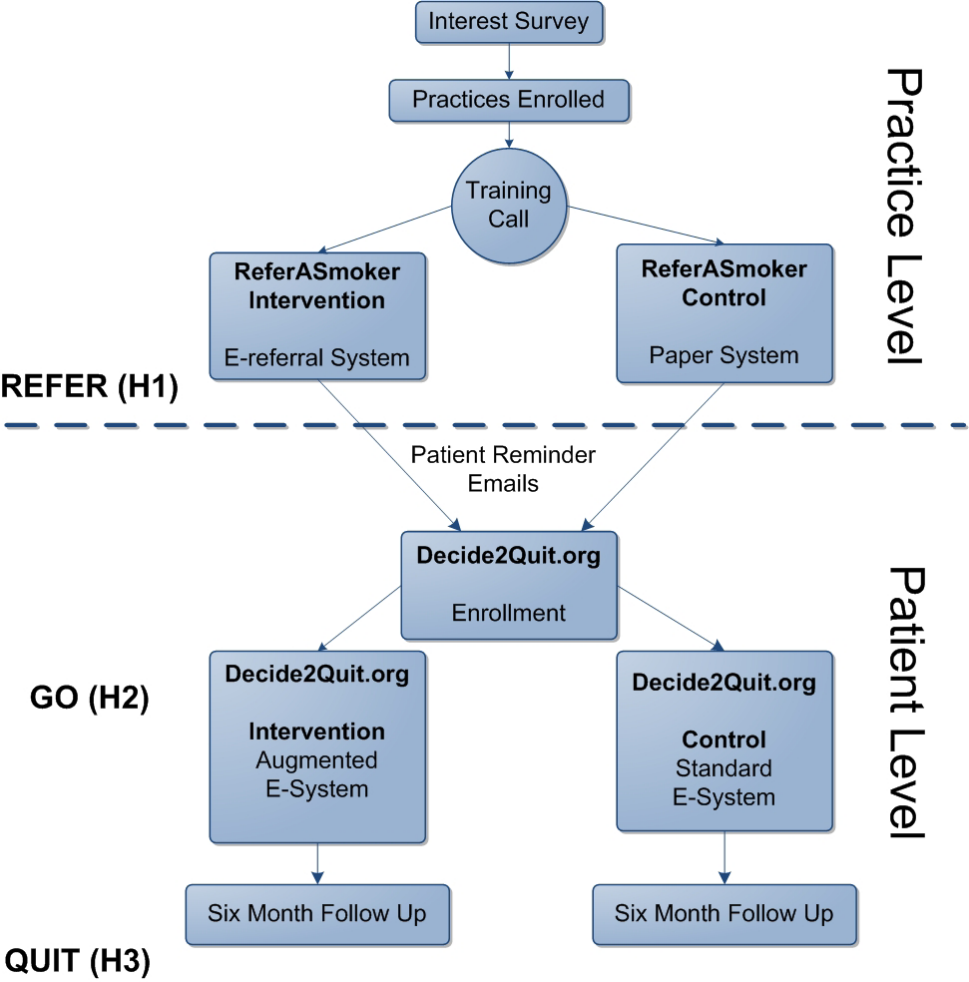
**Enrollment and randomization strategy**.

Thus, our study is a cluster-randomized trial, with patients clustered at the practice level, and a subsequent, within-practice, patient-level randomization (Figure 2). This is further detailed under *Randomization *below.

### Objectives

**Figure 3:**
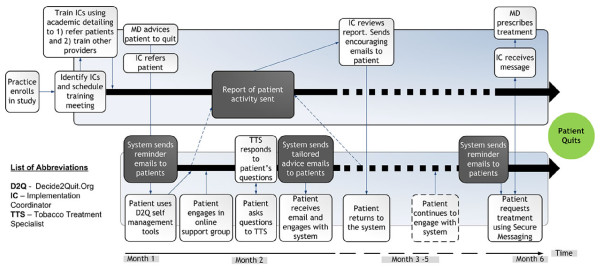
**How the integrated QUIT-PRIMO is conceptualized to improve processes of care (5 *A*s) and increase smoking cessation: use of the clinical microsystem intervention over time by one example practice and one patient**. **A1: Ask**--ReferaSmoker sends email prompts to providers reminding them of the importance of smoking cessation; provider downloads printable chart stickers, etc., to increase systematic screening by nurses. **A2: Advise--**ReferaSmoker materials provide additional knowledge to providers on strong advice; provider advises patient. **A3: Assess--**Provider explains content of Decide2Quit and assesses willingness of patient to use system and to quit. **A4: Assist--**Patient agrees to be recruited and nurse enters patient email into ReferaSmoker online portal and patient is enrolled into the system. Decide2Quit sends email reminders to the patient. Patient uses system and talks to family because of the motivational messages. **A4: Assist--**Patient engages in the online support group, shares his quitting experiences and finds others with similar experiences, posts a question online, and interacts with other smokers trying to quit. **A4: Assist--**Patient selects a tobacco treatment specialist (TTS) and posts a question to her using the MyMail feature of the system. The TTS responds with helpful suggestions, and the patient returns to the system to read her responses. **A4: Assist--**Patient continuously receives tailored "advice" emails from the system. Emails are from experts and peers. **A5: Arrange--**Nurse (and/or physician) reviews reports of patient use and follows up. Nurse sends a template-driven email message encouraging use of the system and offering treatment. **A4: (more) Assist**--Patient returns to Decide2Quit repeatedly, is increasingly motivated, requests treatments → quits.

As conceptualized for one patient in an example practice depicted in Figure 3, the intervention is designed to have a sequence of effects on the process of care within each clinical microsystem. The system has the potential to impact provider behavior (nurses and physicians), processes of care, and patient behavior. Thus, we have designed our main evaluation to assess several key areas of influence, which we have abbreviated as Refer → Go → Quit. To evaluate the impact of the provider and patient intervention, we have proposed the following three hypotheses:

Hypothesis 1 (Refer): More patients will be **Refer**red to the Decide2Quit self-management resource website in the QUIT-PRIMO e-referral practices compared with information prescription practices.

Hypothesis 2 (Go): The proportion of referred patients who **Go **to the patient self-management website due to the QUIT-PRIMO practice proactive e-referrals will be greater compared with the paper information-prescription practices.

Hypothesis 3 (Quit): The proportion of referred smokers who **Quit **at six months will be greater among those in the augmented Decide2Quit.org intervention compared with the standard intervention.

### Outcomes

For our three hypotheses, we have three primary outcome variables (*i.e*., Refer, Go, Quit). For the primary analysis for hypothesis 1, the outcome will be the number of smokers referred from each group transformed into an average count per month by dividing the total by the length of time in months from the practice's first referral. As discussed above, in both arms, practices will use paper information prescriptions. The leave-behind part of the information prescription (see Appendix 1) will allow a consistent measure of referrals. In the QUIT-PRIMO intervention, the server will track the number of electronic referrals, allowing us to compare rates of referral based on paper and server in the intervention arm.

For hypothesis 2, our outcome will be the proportion of those referred who go to the website. Our interest in hypothesis 2, expressed as a proportion, will be the proportion of patients referred who log on, or "Go," to the website (% who go = number who visit/number referred). The number of smokers who visit will be recorded by the Decide2Quit system, linking each visitor to their primary care provider at initial registration. The number referred is continuously registered directly by the leave-behind referral receipts of the information prescriptions.

For hypothesis 3, we will define the outcome in two ways. In both approaches, the numerator will be the number of patients who report cessation at six-month follow-up calls. In our primary intent-to-treat analysis, the denominator will be all smokers who are referred (the same denominator used in hypothesis 2), estimating a population effect. This represents a conservative assessment since we assume that many patients will not go, and for the purposes of analyses, we will assume that these patients will not have quit. As a secondary analysis, we will assign the denominator as the number of patients who go to the website. For this secondary outcome, consistent with current guidelines for smoking cessation trials, we will assume that patients who are lost to follow-up, including those who go and do not agree to follow-up, are smokers [34].

### Sample size

We calculated sample size for each of our three hypotheses. Power was primarily driven by hypothesis 3 (six-month cessation). We first estimated the number of smokers per practice that will participate in the website over time. The average patient panel of a primary care provider is approximately 2,300, although not all are seen in a given year. We estimated 1,500 visits per year. Based on tobacco use prevalence, approximately 22% of the patients will be smokers. To be conservative, we assume that we will have only two providers per practice actively participating in the intervention. Using these numbers, we have estimated the number that will participate per practice yearly (Table 2). We expect 158 referrals from each intervention practice and 79 from each control practice. Based on these samples, and assuming a cessation rate of 10% among patients randomized to the standard Decide2Quit.org, we have 80% power to detect a 5% difference in cessation, comparing the standard and augmented Decide2Quit.org.

**Table 2:** Flow of 3,000^a ^patients through the intervention--Refer → Go → Quit

	Intervention		Control	
**Start with 3,000 patient visits**	**Percent**	**Resulting N**	**Percent**	**Resulting N**

Smokers	22%	660	22%	660

Smokers **REFER**red	24%	158	12%	79

Smokers referred who **GO**	40%	63	20%	16

Smokers that go who **QUIT**	15%	10	10%	2

^a^3,000 patient visits (1,500 per year over two years).

### Randomization

#### Sequence generation and implementation

Practices will be recruited utilizing a mass mailing. An initial interest letter will be sent describing the study and that participation will provide access to tools aimed at enhancing referral of patients to a customized smoking cessation intervention website. Practice eligibility will be determined based on the interest survey, and practices will subsequently be asked to complete a practice consent form and baseline practice survey. With survey and consent returned, our study staff will contact practices and identify and train two implementation coordinators, nurses, or other practice staff who will participate in the referral process. Our staff will talk the implementation coordinators through registration and the referral process. During training, the first implementation coordinator will complete an online consent and survey, followed by randomization.

We developed an online randomization program, based on a block-randomization strategy (a randomization table with blocks of 10) linked to registration. Our statistician, JR, developed the randomization table and only JR and RSS (who developed the randomization system) have access to the table. When the first user from a practice is randomized, the system will look up the next allocation in the randomization table and the user-provider then has access to the intervention or comparison version of ReferaSmoker.org. All subsequent providers from the sample practice will then be randomized to the same arm.

Patients will also be randomized to the standard Decide2Quit.org or augmented Decide2Quit.org using the online randomization program but using a separate randomization table, also generated by JR.

### Blinding (masking)

Because each practice is informed, they will be provided tools aimed at enhancing referral, and since specifics of either arm are not described at any time, the practice is blind to group assignment. During the training process up to the point of randomization, the study coordinator is blind to which arm the practice will be assigned; however, they are unblinded once randomization occurs in order to provide direction for the appropriate referral process. Each study coordinator is trained to minimize any bias in communication with the implementation coordinators based on which arm they are assigned. Practices remain blind. In turn, all patients are blind to website characteristics and which randomized group they will be assigned to. Patients remain blind until completion of the study. At completion of the study, all practices and patients are unblinded and given the opportunity to utilize all features of both websites.

### Analysis

The mean number of referrals per month will be compared by study group using a two-sided *t*-test, employing Satterthwaite's approximation if the variances are substantially different. We will also examine the distribution of the referrals per month by study group and will use the two-sided Wilcoxon test if they appear non-Gaussian. We will assess the adequacy of randomization on characteristics of the practice that might influence referral rates. We will have data from an administrative database (size of practice, number of providers and staff) and provider reports of proportion of smokers in the practice. We will also conduct adjusted (using Poisson or negative binomial models based on distribution) analyses accounting for these factors.

For hypothesis 2, whether or not a patient goes will be considered a dichotomous outcome in a patient-level analysis. The main patient-level analysis will use a generalized linear model with a logit link to evaluate whether a referred smoker goes to the patient website. As this hypothesis represents a cluster-randomized trial, because each website links patients back to their practice, we will use generalized estimating equation (GEE) methods to account for clustering within practices.

For hypothesis 3, patients will be randomized within practices to standard Decide2Quit and augmented Decide2Quit. Because we are comparing rates in all instances between the standard Decide2Quit system and the augmented Decide2Quit, we will use a two-group chi-square test of equal proportions to test the statistical difference between the quit rates. We will next use a generalized linear model with a logit link to model tobacco cessation by treatment assignment adjusted for baseline readiness to change as entered into the website by the patient.

## Discussion

Despite some success in targeting one aspect or another of health services, single-dimension provider or patient implementation strategies are inherently limited. Using the Internet as a delivery method provides the potential to link multiple provider and patient intervention components, but this potential has not yet been realized. Our goal is to link providers and patients through an innovative electronic system with redundant cues and reminders to encourage participation at all levels of the clinical microsystem. Within the intervention group, the system will provide the practices with feedback and the patients with encouragement.

Once referred, the patient system has been developed to include current innovations in online smoking cessation interventions. Standard components include educational materials, interactive decision support tools (*e.g*., "What do I have to overcome?"-an assessment of triggers to smoking), and a quit plan tailored to readiness to quit. Additional, more innovative components, including online counseling with a tobacco treatment specialist through an asynchronous secure messaging system, are available in the augmented Decide2Quit.org. Our goal is to analyze the impact of this integrated system in terms of process (Refer and Go) and outcomes (six-month smoking cessation).

## Competing interests

The authors declare that they have no competing interests.

## Authors' contributions

TKH, the principal investigator of the study, conducted the data analysis with the oversight of JR, drafted the initial manuscript, and reviewed and approved the final draft. RSS developed the figures and wrote part of the manuscript. RSS, JR, DEF, MNR, and JJA participated in study design and data collection and critically reviewed, edited, and approved the final draft.

## Supplementary Material

Additional File 1 **Information prescription sheet for the Intervention**. A copy of the information prescription that will be provided to the Intervention practices. This sheet contains a space for the patient's email, provider signature, and the smoking cessation website address (Decide2Quit.org). Click here for file

Additional File 2 **Information prescription sheet for the practice-level comparison arm**. A copy of the information prescription that will be provided to the Comparison practices. This sheet does not contain a space for the patient's email denoting the difference between the intervention and comparison arm referral process. Click here for file
