# Supplementary material for: The QUIT-PRIMO provider-patient Internet-delivered smoking cessation referral intervention: a cluster-randomized comparative effectiveness trial: study protocol
Source: Implement Sci. 2010 Nov 17;5:87. doi: 10.1186/1748-5908-5-87 (PMC2998448; doi:10.1186/1748-5908-5-87)
Supplement: Additional File 2 — Information prescription sheet for the practice-level comparison arm. A copy of the information prescription that will be provided to the Comparison practices. This sheet does not contain a space for the patient's email denoting the difference between the intervention and comparison arm referral process. [file 1748-5908-5-87-S2.PDF]

PROVIDER

ADVISE smoker to stop smoking. Tell your patient: ***"It is important that you quit smoking now, and I can help you."***

☐ **ASSESS** readiness to quit: Ask the patient, ***"Are you seriously thinking about quitting smoking within the next 6 months?"*** ☐ Yes ☐ No

☐ **ASSIST** smoker to quit: ☐ Brief counseling ☐ Prescription medications if appropriate:

Nicotine replacement (CIRCLE) patch gum lozenge inhaler nasal spray

(CIRCLE) Buproprion (Zyban or Wellbutrin SR) Chantix Other

☐ **ARRANGE** follow-up: ☐ Information Rx given to patient

PATIENT

DECIDE 2QUIT

Information Prescription

Rx

Patient Instructions: Your healthcare provider has referred you to **www.decide2quit.org**

Type this into the address bar of your web browser. Once you log in, you will receive :

- Interactive calculator and education materials to help you think about smoking and quitting
- How to get support from those around you (friends, family, doctor)

____________________________ ____________________________

Referring Physician/Nurse Date

AGREEMENT

I (undersigned) understand that quitting smoking is the single most important thing I can do for my health. I know that quitting smoking will not be easy but I have the support of my physician/nurse to be successful. I agree to be referred and will consider visiting the www.decide2quit.org website.

Patient Signature ____________________________ Date ____________________

Decide2Quit HELPDESK • Tel: (508) 856-3543
